# High Positive Correlations between *ANRIL* and *p16*-*CDKN2A*/*p15*-*CDKN2B*/*p14*-*ARF* Gene Cluster Overexpression in Multi-Tumor Types Suggest Deregulated Activation of an *ANRIL–ARF* Bidirectional Promoter

**DOI:** 10.3390/ncrna5030044

**Published:** 2019-08-21

**Authors:** Kinan Drak Alsibai, Sophie Vacher, Didier Meseure, Andre Nicolas, Marick Lae, Anne Schnitzler, Walid Chemlali, Jerome Cros, Elisabeth Longchampt, Wulfran Cacheux, Geraldine Pignot, Celine Callens, Eric Pasmant, Yves Allory, Ivan Bieche

**Affiliations:** 1 Platform of Experimental Pathology, Institut Curie, 75248 Paris, France; 2 Unit of Pharmacogenomics, Department of Genetics, Institut Curie, 75248 Paris, France; 3 Department of Diagnostic and Theranostic Medicine, Institut Curie, 75248 Paris, France; 4 Department of Pathology, Beaujon Hospital, APHP Nord, 92110 Clichy, France; 5 Department of Pathology, Foch Hospital, 92150 Suresnes, France; 6 Department of Genetics, Cochin Hospital, APHP, 75014 Paris, France; 7 Cochin Institute, Inserm U1016, Paris Descartes University, 75014 Paris, France

**Keywords:** lncRNA, *ANRIL* overexpression, *p16-CDKN2A/p15-CDKN2B/p14-ARF* cluster

## Abstract

The *CDKN2B-AS1* gene, also called *ANRIL*, is located at the human *CDKN2A/B* locus at 9p21.3 and transcribed by RNA polymerase II into a long non-coding RNA of 3834 bp. The *CDKN2B-AS1* gene overlaps a critical region of 125 kb covering the *CDKN2B* gene. The *CDKN2A/B* locus encompasses three major tumor suppressors juxtaposed and joined into a *p16-CDKN2A/p15-CDKN2B/p14-ARF* gene cluster. *CDKN2A* encodes splice variants p16-CDKN2A and p14-ARF, and *CDKN2B* encodes p15-CDKN2B. *ANRIL* shares a bidirectional promoter with the *p14-ARF* gene and is transcribed from the opposite strand to the cluster. We performed an analysis of the expression level of *ANRIL* and tumor suppressor *p16-CDKN2A, p15-CDKN2B*, and *p14-ARF* genes using quantitative RT-PCR in a multitumor panel. We observed the overexpression of the four genes *ANRIL*, *p16-CDKN2A, p15-CDKN2B*, and *p14-ARF* in the great majority of the 17 different cancer types. *ANRIL* was upregulated in 13/17 tumors compared to normal tissues, ranging from 5% (prostate cancer) to 91% (cervix cancer), with variable expression of *p16-CDKN2A, p15-CDKN2B*, and *p14-ARF* genes. A high positive correlation was identified between levels of expression of *ANRIL* and the three tumor suppressors. The strongest positive association was observed with *p14-ARF* (*p* < 0.001) in all but one (lung squamous cell carcinoma) of the examined tumor types. This correlation suggests coordinated deregulated mechanisms in all cancer types through aberrant activation of a bidirectional *p14-ARF/ANRIL* promoter. Furthermore, significant positive correlation was unexpectedly established in prostatic carcinomas, in contradiction with previous data.

## 1. Introduction

**Figure 1:**
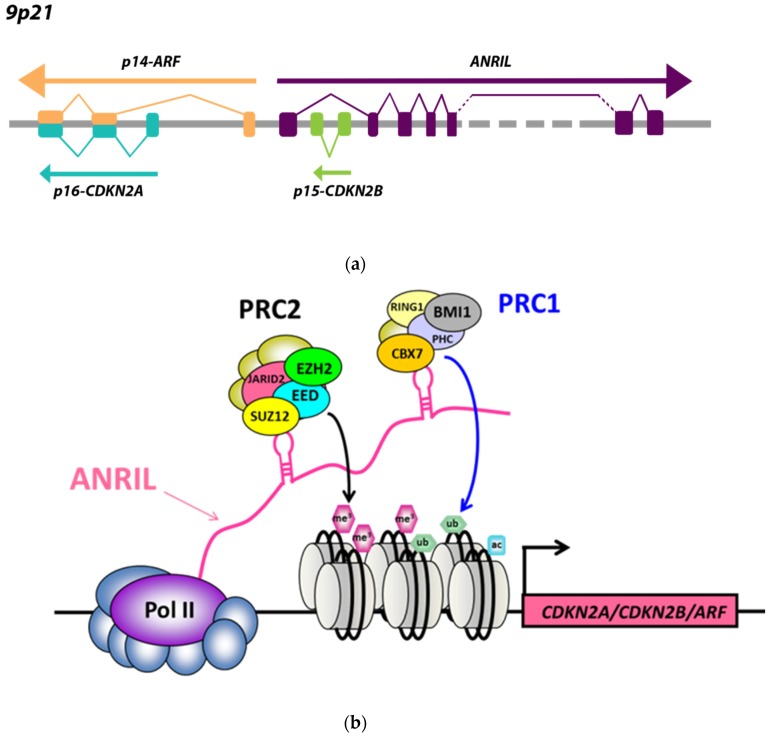
(**a**) *ANRIL–ARF* bidirectional promoter; (**b**) Interactions between the long non-coding RNA (lncRNA) *ANRIL* and polycomb PRC2/PRC1 repressive complexes at the *p16-CDKN2A/p15-CDKN2B/p14-ARF* locus (see Supplementary Table S2).

The *CDKN2B-AS1* gene, also called *ANRIL*, is located at the human *CDKN2A/B* locus at 9p21.3, and is transcribed by RNA polymerase II into a long non-coding RNA of 3834 bp. The *CDKN2B-AS1* gene contains 21 reported exons over a critical region of 125 kb covering the *CDKN2B* gene [1]. This long non-coding RNA (lncRNA) includes LINE, SINE, and Alu repetitive sequences, and comprises numerous linear and circular splice variants [2]. The *CDKN2A/B* locus encompasses three major tumor suppressors juxtaposed and joined into a *p16-CDKN2A/p15-CDKN2B/p14-ARF* gene cluster [1]. *CDKN2A* encodes splice variants p16-CDKN2A and p14-ARF, and *CDKN2B* encodes p15-CDKN2B (Figure 1a). *ANRIL* shares a bidirectional promoter with the *p14-ARF* gene, and is transcribed from the opposite strand to the cluster [1] (Figure 1a). Expression of these two genes is therefore coordinated, and activation of this divergent promoter is provided by the transcription factor E2F1 [3,4]. *ANRIL* intervenes mainly at the transcriptional level through epigenetic mechanisms. This lncRNA acts mainly in *cis* at the *p16-CDKN2A/p15-CDKN2B/p14-ARF* cluster, whose three tumor suppressor genes are involved in stem cell renewal, senescence, and apoptosis by promoting anti-proliferative and pro-apoptotic activities of Rb1 and p53 [5]. *ANRIL* functions as a docking platform for the polycomb complexes PRC1 and PRC2, which are epigenetic regulators acting in coordination to modify the histone code [6,7,8] (Figure 1b). The PRC2 complex mainly comprises subunits JARID2, EED, SUZ12, and EZH2, which maintain chromatin repression by catalyzing mono-, di-, and trimethylation of histone H3 lysine 27 (H3K27me1, H3K27me2, and H3K27me3) [9]. The PRC1 complex is composed of several subunits, such as PHC, CBX7, BMI1, and RING1a/1b, which catalyze the mono-ubiquitination of H2A on K119 (H2AK119ub1), so as to firmly maintain chromatin inactivation [10]. During this process, *ANRIL* promotes specific interactions with the two proteins SUZ12 (PRC2) and CBX7 (PRC1), in order to stabilize PRC1/PRC2 complexes, modify the histone code of the *p16-CDKN2A/p15-CDKN2B/p14-ARF* locus, and ensure a firm and perennial transcriptional repression [5]. *ANRIL* is overexpressed in many malignant tumors, including leukemias and stomach, prostate, kidney, esophagus, colon, breast, ovary, and lung carcinomas [6,11,12,13,14,15]. In these cancers, statistical studies and meta-analyses have revealed that the overexpression of *ANRIL* was positively correlated with advanced TNM stage and occurrence of lymph node metastases, and constituted an independent predictor of adverse overall survival [16]. Mounting data from the literature suggests that this lncRNA could function as an oncogenic driver, consistent with the association of multiple single nucleotide polymorphisms (SNPs) in 9p21.3 with numerous cancers [17]. Structural alterations of *ANRIL* (deletions and translocations) were described in neurofibromas and gliomas [18], and *MTAP-ANRIL* fusion transcripts were observed in melanomas [19]. During carcinogenesis, DNA alterations favor activation of the ATM-E2F1 pathway and induce *ANRIL* dysregulation, resulting in its aberrant chronic overexpression. Aberrant *ANRIL* overexpression causes inefficiency of DNA damage repair mechanisms, leading to genomic instability and tumor progression [20] via cell cycle progression, inhibition of apoptosis and senescence, tumor proliferation, and angiogenesis [21]. A study on human prostatic tumors suggested that *ANRIL* overexpression was mainly accompanied by transcriptional inactivation of the *p16-CDKN2A/p15-CDKN2B/p14-ARF* locus through *cis* direct interaction with the two repressive polycomb complexes PRC2 and PRC1 [6]. However, little is known about the mechanisms of *ANRIL* deregulation in most other types of cancers. In a recent article concerning a series of 456 breast carcinomas, we identified an unexpected strong positive link between *ANRIL* and the *p16-CDKN2A/p15-CDKN2B/p14-ARF* locus and a complex pattern of interactions between *ANRIL*, PRC2/PRC1, and several suppressive/oncogenic miRNAs [15]. We identified an altered repressive function of PRC2 and PRC1 at RNA and protein levels, resulting in the absence of *p16-CDKN2A/p15-CDKN2B/p14-ARF* cluster inactivation with frequent *p16-CDKN2A*, *p15-CDKN2B*, and *p14-ARF* overexpressions. This discrepancy between prostate and breast cancer suggests a large variability of the *ANRIL/p16-CDKN2A/p15-CDKN2B/p14-ARF* interaction network depending on the tumor type. Here, we analyzed expression levels of *ANRIL* and the *p16-CDKN2A/p15-CDKN2B/p14-ARF* locus in a multi-tumor panel of 702 malignant tumor samples among 17 different types of cancers.

## 2. Materials and Methods

In this study, we investigated the expression of *ANRIL* and *p16-CDKN2A/p15-CDKN2B/p14-ARF* gene cluster by quantitative RT-PCR in a multi-tumor panel of different types of cancers.

### 2.1. Patients and Samples

Samples from 17 different types of primary tumors (total, *n* = 702; Supplementary Table S1) were obtained from multi-center Departments of Pathology from 1978 to 2010. All patients were informed that their tumor samples might be used for scientific purposes, and had the opportunity to decline. Since 2007, patients treated in our institutions have given their approval by signed informed consent. This study followed institutional guidelines as put forth by the French Ethical Committee and the Ethics Committee of Curie institute (Agreement number C75-05-18). Samples were immediately stored in liquid nitrogen until RNA extraction and then stored at −80 °C. A tumor sample was considered suitable for this study if the proportion of tumor cells exceeded 70%. Normal tissues belong to non-cancerous patients (e.g., mammoplasties for breast tissues) or adjacent normal tissues from cancer patients (distance >1 cm from tumor) for which a morphological analysis has eliminated a micrometastatic disease that was confirmed by combining an immunohistochemical study that has revealed a Ki67 index of epithelial cells of <2% and a RT-PCR analysis using MKI67 gene expression.

### 2.2. RNA Extraction

Total RNA was extracted by using acid-phenol guanidium, as previously described [22]. RNA quality was determined by electrophoresis through agarose gels, staining with ethidium bromide, and visualization of the 18S and 28S RNA bands under ultraviolet light. Determination of RNAs’ integrity is based on combination of three criteria: the 28S/18S ratio, the RIN (RNA integrity number) value, and the electrophoretic profile. A qualitative assessment of the type “Good, Medium, or Bad” was attributed for each of the three criteria: 28S/18S ratio (≥1.5, Good; 1.3 < ratio < 1.5, Medium; <1.3, Bad), RIN value (≥7, Good; 6 < RIN < 7, Medium; <6, Bad), and electrophoretic profile (flat baseline and RIN >7, Good; high baseline in the “fast region”, Medium; relatively high baseline, Bad). We selected RNAs with high integrity suitable for this study when a minimum of two out of three criteria were classed.

### 2.3. Real-Time RT-PCR

*ANRIL*, *p16-CDKN2A*, *p15-CDKN2B*, and *p14-ARF* gene mRNA expression levels were quantified using real-time RT-PCR. Quantitative values were obtained from the cycle number (*C*_t_ value) at which the increase in the fluorescence signal associated with exponential growth of PCR products started to be detected by the laser detector of the ABI Prism 7900 Sequence Detection System (Perkin-Elmer Applied Biosystems), using PE Biosystems analysis software according to the manufacturer’s manuals. The precise amount of total RNA added to each reaction mix (based on optical density) and its quality (i.e., lack of extensive degradation) are both difficult to assess. We therefore also quantified transcripts of the *TBP* gene (Genbank accession: NM_003194) encoding the TATA box-binding protein (a component of the DNA-binding protein complex TFIID) as an endogenous RNA control, and normalized each sample on the basis of its *TBP* content. We selected *TBP* as an endogenous control because the prevalence of its transcripts is moderate and because there are no known TBP retropseudogenes (retropseudogenes lead to coamplification of contaminating genomic DNA and thus interfere with RT-PCR, despite the use of primers in separate exons). We also selected *RPLP0* because the prevalence of its transcripts is high as compared with *TBP* and because this gene is used widely as an endogenous control for Northern blot analysis (known better as 36B4). Results, expressed as *N*-fold differences in target gene expression relative to the *TBP* (or *RPLP0*) gene and termed “*N*_target_,” were determined as *N*_target_ = 2^Δ*C*t_*sample*^, where the Δ*C*_t_ value of the sample was determined by subtracting the average *C*_t_ value of the target gene from the average *C*_t_ value of the *TBP* (or *RPLPO*) gene. The smallest amount of mRNA that was detectable and quantifiable by RT-qPCR (ΔCt = 35) was used as a reference (basal mRNA level = 1) to normalize the data for normal and tumoral tissue samples. For each tumor type, the *N*_target_ values of the samples were also subsequently normalized such that the median of the *N*_target_ values for each normal tissue type was 1. *N*_target_ values of 0.33 or less were considered to represent under-expression, and values of 3 or more to represent overexpression of these genes in tumor samples. We previously used the same approach to determine cut-off points for the altered expression of tumor genes [15]. The primers for *TBP*, *RPLPO*, *ANRIL*, *p16-CDKN2A*, *p15-CDKN2B*, and *p14-ARF* genes were chosen with the assistance of the Oligo 6.0 program (National Biosciences). We scanned the dbEST and nr databases to confirm the total gene specificity of the nucleotide sequences chosen for the primers and the absence of SNPs. To avoid amplification of contaminating gDNA, one of the two primers was placed at the junction between two exons or on two different exons. The primer pairs for each *CDKN2A/B* cluster genes were selected to be unique when compared to the sequences of the two other *CDKN2A/B* cluster genes—in particular, comparison between *p14ARF/CDKN2A* and *p16/CDKN2A*. Moreover, to avoid amplification of contaminating genomic DNA, one of the two primers was placed in a different exon. For example, the upper primer of *TBP* was placed at the junction between exons 5 and 6, whereas the lower primer was placed in exon 6. The nucleotide sequences of the oligonucleotide hybridization primers are shown in Supplementary Table S2. Agarose gel electrophoresis was used to verify the specificity of PCR amplicons. The conditions of cDNA synthesis and PCR were as described [23]. QPCRs were performed with duplicates for each data point.

### 2.4. Database Sources

RNA Sequencing data and mRNA expression levels in the TCGA series used for statistical analysis between *ANRIL*, *p16-CDKN2A*, *p14-ARF*, and *p15-CDKN2B* were downloaded from the publicly available database cBioPortal for Cancer Genomics (https://www.cbioportal.org). Graphical view of the *ANRIL (CDKN2B-AS1)* gene was adapted from the University of California Santa Cruz (UCSC) Genome Browser on Human Dec. 2013 (GRCh38/hg38) Assembly. RNA and protein expressions overviews of p14-ARF, p15-CDKN2B, and p16-CDKN2A in various types of cancer and protein expression of p14-ARF, p15-CDKN2B, and p16-CDKN2A in prostate cancer TMA samples were adapted from Protein Atlas website—version 18.1 (https://www.proteinatlas.org).

### 2.5. Statistical Analysis

Relationships between mRNA levels of the different target genes were analyzed using the nonparametric Spearman rank correlation test (relation between two quantitative parameters). Differences were considered significant at confidence levels greater than 95% (*p* < 0.05).

## 3. Results

**Figure 2:**
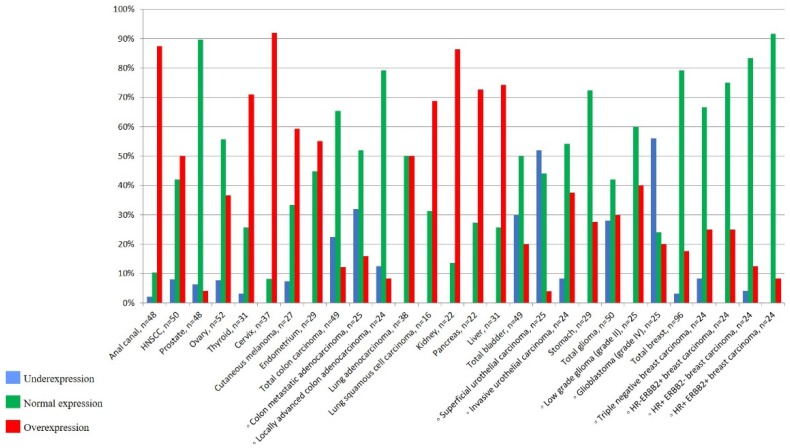
Percentage of underexpression, normal expression, and overexpression of *ANRIL* in tumor tissues.

**We identified the overexpression of *ANRIL* in 13 among the 17 cancer types compared to normal tissues.** Interestingly, the expression status of *ANRIL* was different depending on histological subtype, tumor stage, and tumor grade. *ANRIL* overexpression varied according to histological subtype of malignant tumors from 5% (prostate) to 91% (cervix), and was predominant in anal canal (88%), kidney (87%), and liver (74%) carcinomas (Figure 2, Supplementary Figure S1). *ANRIL* upregulation was more frequent in organs that mainly develop carcinomas of squamous cell type, such as cervix (91%) and anal canal (88%). Accordingly, overexpression of *ANRIL* in lung cancer was more marked in squamous cell carcinomas (68%) than adenocarcinomas (50%). In colon carcinomas, *ANRIL* overexpression was observed in invasive tumors (12%) compared to normal tissue (0%), and was higher in carcinomas at metastatic stage (16%) compared to locally invasive primary colon carcinomas (8%). In bladder carcinomas, *ANRIL* upregulation was more pronounced in invasive carcinomas (38%) than in superficial non-invasive urothelial tumors (4%). In invasive breast carcinomas, *ANRIL* overexpression was higher in triple-negative (25%) and HER2+ (24%) subtypes than in luminal carcinomas (12%).

Different profiles of expression of the three tumor suppressor genes *p16-CDKN2A*, *p15-CDKN2B*, and *p14-ARF* were observed in the multi-tumor panel. *P16-CDKN2A* was mostly overexpressed in cervix (100%), anal canal (83%), and ovary (81%) (Supplementary Figure S2A,B). *P15-CDKN2B* was mainly upregulated in pancreas (91%), thyroid (78%), and kidney (77%) carcinomas, whereas *p15-CDKN2B* underexpression was identified in colon (90%) carcinomas, skin melanomas (59%), and head and neck squamous cell carcinoma (HNSCC; 38%) (Supplementary Figure S2C,D). *P14-ARF* was overexpressed in all tumors, mainly in kidney (100%), cervix (95%), and anal canal (94%) carcinomas (Supplementary Figure S2E,F). mRNA levels calculated as described in the Materials and Methods showed abundance of the target relative to the endogenous control (*TBP*), to normalize the starting amount and quality of total RNA. Similar results were obtained with the second endogenous control, *RPLP0* (data non shown).

A very high positive correlation was identified between *ANRIL* and the *p16-CDKN2A/p15-CDKN2B/p14-ARF* gene cluster levels of expression in the vast majority of tumors as compared to normal tissues. Most normal tissues showed no or few significant associations between *ANRIL* and the *p16-CDKN2A/p15-CDKN2B/p14-ARF* gene cluster, and marked positive correlation values (*p* < 0.001) were only found between *ANRIL* and *p14-ARF* in head and neck (*p* = 0.000026) and colon (*p* = 0.00014); to a lesser degree between *ANRIL* and *p15-CDKN2B* in anal canal (*p* = 0.024), liver (*p* = 0.027), and lung (*p* = 0.0013); and between *ANRIL* and *p16-CDKN2A* in ovary (*p* = 0.019) (Table 1). Conversely, most tumors revealed a strong positive correlation between *ANRIL* and the *p16-CDKN2A/p15-CDKN2B/p14-ARF* gene cluster levels of expression, especially HNSCC, ovary, thyroid, cervix, colon, kidney, bladder, brain, and breast (*p* < 0.0000001) (Table 2). The strongest positive correlation was observed with the tumor suppressor *p14-ARF*. All examined tumors except lung squamous cell carcinoma revealed a very high positive association between *ANRIL* and *p14* expression, as compared with *p15-CDKN2B* and *p16-CDKN2A*. No negative association was identified, particularly in prostatic carcinomas. We confirmed our experimental results by performing an in-silico analysis of mRNA expression levels in the TCGA series (RNA sequencing data from https://www.cbioportal.org), which showed positive correlation between *ANRIL* and *p14-ARF* and *p16-CDKN2A* (*p* < 0.0001), and between *ANRIL* and *p15-CDKN2B* (*p* < 0.0001) in both breast carcinomas and prostate carcinomas (Supplementary Table S3).

## 4. Discussion

Concerning *CDKN2A-B* locus deregulation in cancers, literature data provided contradictory results. Against commonly accepted *ANRIL*- and PRC2/PRC1-induced epigenetic inactivation of the three oncosuppressors, positive correlations between *ANRIL, p16-CDKN2A*, and *p15-CDKN2B* genes were frequently identified in numerous tissues and non-cancerous diseases [24,25,26,27,28]. In accordance with these data, in our multitumor panel we observed overexpression of the four genes *ANRIL*, *p15-CDKN2B, p16-CDKN2A*, and *p14-ARF* in a great majority of the 17 tumor types. Although most normal tissues showed non-significant association between the expression of *ANRIL* and genes belonging to the *p16-CDKN2A/p15-CDKN2B/p14-ARF* gene cluster, high positive correlations were identified between *ANRIL* and *p16-CDKN2A, p15-CDKN2B*, and *p14-ARF* genes in the great majority of malignant tumors, suggesting coordinated alterations of their regulation in all types of cancers. Furthermore, in agreement with the presence of an E2F1-activated divergent promoter shared between *ANRIL* (*CDKN2B-AS1)* and *p14-ARF* genes, we observed the strongest positive correlation between *ANRIL* and the tumor suppressor *p14-ARF*, and to a lesser degree with *p15-CDKN2B* and *p16-CDKN2A*. This very significant positive association is in favor of a mechanism of deregulation in cancer involving the aberrant activation of a bidirectional *ANRIL–p14-ARF* promoter. Moreover, a positive correlation was unexpectedly observed in prostatic carcinomas of our series between *ANRIL* and *p14-ARF*, *p15-CDKN2B*, and *p16-CDKN2A*. These results are contradict previous data from the literature suggesting that in prostatic carcinomas *ANRIL* overexpression was mainly accompanied by transcriptional inactivation of the *p16-CDKN2A/p15-CDKN2B/p14-ARF* locus via repressive action of the two polycomb complexes PRC2 and PRC1 [6]. Our experimental and in-silico results are in accordance with recent data showing transcriptional co-activation of *ANRIL, p14-ARF, p15-CDKN2B*, and *p16-CDKN2A* in cancers induced by a bidirectional *ANRIL–CDKN2A* promoter in cell lines of colon cancer [29]. Despite important progress, *ANRIL* deregulation in cancer remains largely unknown, and only a few mechanisms have recently been evocated, such as *ANRIL* and *p16-CDKN2A* promoter methylation status regulating *ANRIL/p16-CDKN2A* and *ANRIL/p14-ARF* transcription [29,30] and transcription factors activating *ANRIL* transcription [31,32,33]. Splicing was recently implicated in *ANRIL* deregulation, and *ANRIL* transcript stability seems to be influenced by miRNAs and proteins [34,35]. *ANRIL–*miRNAs and PRC2/PRC1–miRNAs interacting networks’ deregulation has also been implicated in carcinogenesis [36,37,38].

In conclusion, the lncRNA *ANRIL* is an oncogenic driver that is deregulated in most cancers, and a better knowledge of its mechanisms of deregulation is a pivotal step in confirming its therapeutic potential.

## Figures and Tables

**Table 1:** Correlations between ANRIL and P14, P15 and P16 mRNA expression levels in the series of normal tissues.

Normal Tissues	Nbr	*P14*		*P16*		*P15*	
		r	*p*-value *^a^*	r	*p*-value *^a^*	r	*p*-value *^a^*
**Anal canal**	17	+0.297	0.25 (NS)	+0.385	0.12 (NS)	+0.542	0.024
**Head and neck**	27	**+0.727**	**0.000026**	+0.137	0.50 (NS)	+0.159	0.43 (NS)
**Prostate**	7	+0.094	0.83 (NS)	−0.668	0.10 (NS)	+0.256	0.58 (NS)
**Ovary**	27	+0.423	0.026	+0.447	0.019	+0.010	0.96 (NS)
**Thyroid**	9	+0.852	0.0038	−0.248	0.53 (NS)	−0.201	0.61 (NS)
**Cervix**	14	+0.534	0.047	−0.295	0.31 (NS)	+0.116	0.69 (NS)
**Skin**	9	+0.438	0.24 (NS)	+0.479	0.19 (NS)	−0.114	0.77 (NS)
**Endometrium**	8	+0.286	0.50 (NS)	+0.218	0.61 (NS)	+0.571	0.14 (NS)
**Colon**	30	**+0.647**	**0.00014**	+0.209	0.27 (NS)	−0.126	0.51 (NS)
**Lung**	16	+0.644	0.0069	+0.433	0.091 (NS)	+0.732	0.0013
**Kidney**	18	+0.058	0.81 (NS)	+0.073	0.77 (NS)	+0.124	0.63 (NS)
**Pancreas**	11	+0.609	0.045	+0.202	0.56 (NS)	+0.212	0.54 (NS)
**Liver**	10	+0.547	0.099 (NS)	+0.291	0.42 (NS)	+0.688	0.027
**Bladder**	14	+0.459	0.096 (NS)	+0.034	0.90 (NS)	+0.341	0.23 (NS)
**Stomach**	11	+0.361	0.28 (NS)	−0.391	0.23 (NS)	+0.267	0.43 (NS)
**Brain**	21	+0.619	0.0028	+0.419	0.056 (NS)	+0.366	0.099 (NS)
**Breast**	11	−0.136	0.69 (NS)	−0.195	0.57 (NS)	−0.027	0.93 (NS)

^a^ Spearman’s rank correlation.

**Table 2:** Statistical analysis and correlation between *ANRIL* and *P14*, *P15* and *P16* mRNA expression levels in the series of tumoral tissues.

Tumoral Tissues	Nbr	*P14*		*P16*		*P15*	
		r	*p*-value *^a^*	r	*p*-value *^a^*	r	*p*-value *^a^*
**Anal canal**	48	+0.571	0.000037	+0.413	0.0036	+0.247	0.087 (NS)
**HNSCC**	50	+0.709	**<0.0000001**	+0.609	0.000006	+0.667	0.00000042
**Prostate**	48	+0.467	0.00093	+0.427	0.0026	+0.059	0.69 (NS)
**Ovary**	52	+0.728	**<0.0000001**	+0.581	0.000013	+0.502	0.0002
**Thyroid**	31	+0.854	**<0.0000001**	+0.459	0.0092	+0.690	0.000027
**Cervix**	37	+0.843	**<0.0000001**	+0.563	0.00035	+0.548	0.00053
**Cutaneous melanoma**	27	+0.797	0.0000013	+0.392	0.041	+0.854	**<0.0000001**
**Endometrium**	29	+0.801	0.00000042	+0.742	0.000007	+0.634	0.00027
**Total colon carcinoma**	49	+0.832	**<0.0000001**	+0.095	0.52 (NS)	+0.664	0.00000062
**Lung adenocarcinoma**	38	+0.553	0.0003	+0.325	0.047	+0.493	0.0016
**Lung squamous cell carcinoma**	16	+0.380	0.15 (NS)	+0.398	0.13 (NS)	+0.332	0.21 (NS)
**Kidney**	22	+0.947	**<0.0000001**	+0.700	0.00033	+0.363	0.093 (NS)
**Pancreas**	22	+0.684	0.0005	+0.571	0.0054	+0.801	0.000011
**Liver**	31	+0.642	0.00013	-0.292	0.11 (NS)	+0.340	0.058 (NS)
**Total bladder**	49	+0.865	**<0.0000001**	+0.727	**<0.0000001**	+0.762	**<0.0000001**
**Stomach**	29	+0.733	0.00001	+0.219	0.25 (NS)	+0.426	0.02
**Total glioma**	50	+0.902	**<0.0000001**	+0.858	**<0.0000001**	+0.812	**<0.0000001**
**Total breast**	96	+0.758	**<0.0000001**	+0.576	**<0.0000001**	+0.576	**<0.0000001**

^a^ Spearman’s rank correlation.

## Supplementary Materials

The supplementary materials are available online at https://www.mdpi.com/2311-553X/5/3/44/s1.
