# Supplementary material for: High Positive Correlations between ANRIL and p16-CDKN2A/p15-CDKN2B/p14-ARF Gene Cluster Overexpression in Multi-Tumor Types Suggest Deregulated Activation of an ANRIL–ARF Bidirectional Promoter
Source: Noncoding RNA. 2019 Aug 21;5(3):44. doi: 10.3390/ncrna5030044 (PMC6789474; doi:10.3390/ncrna5030044)
Supplement: Supplementary file 1 [file ncrna-05-00044-s001.zip › Supplemental Figure 2C ANRIL Drak Alsibai et al.pptx]

## Slide 1
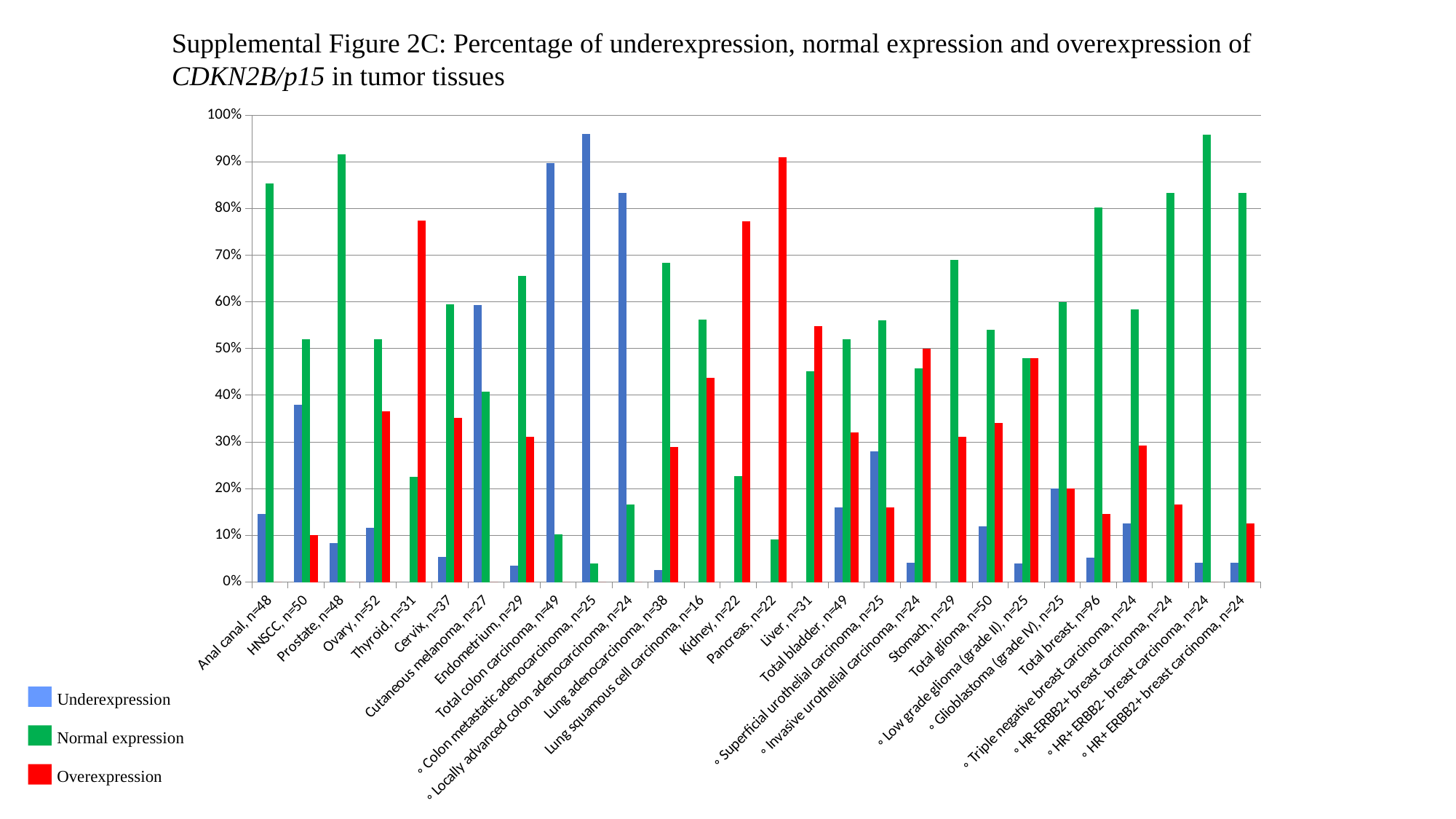

Supplemental Figure 2C: Percentage of underexpression, normal expression and overexpression of CDKN2B/p15 in tumor tissues
### Chart
| Category | Underexpression | % expr normale | Overexpression |
|---|---|---|---|
| Anal canal, n=48 | 0.14583333333333345 | 0.8541666666666665 | 0.0 |
| HNSCC, n=50 | 0.38000000000000017 | 0.52 | 0.1 |
| Prostate, n=48 | 0.08333333333333334 | 0.9166666666666665 | 0.0 |
| Ovary, n=52 | 0.11538461538461539 | 0.5192307692307688 | 0.365384615384616 |
| Thyroid, n=31 | 0.0 | 0.22580645161290333 | 0.7741935483870968 |
| Cervix, n=37 | 0.054054054054054085 | 0.5945945945945946 | 0.35135135135135137 |
| Cutaneous melanoma, n=27 | 0.5925925925925927 | 0.4074074074074076 | 0.0 |
| Endometrium, n=29 | 0.034482758620689655 | 0.655172413793104 | 0.3103448275862071 |
| Total colon carcinoma, n=49 | 0.8979591836734696 | 0.10204081632653061 | 0.0 |
| ◦ Colon metastatic adenocarcinoma, n=25 | 0.9600000000000003 | 0.040000000000000056 | 0.0 |
| ◦ Locally advanced colon adenocarcinoma, n=24 | 0.8333333333333337 | 0.16666666666666663 | 0.0 |
| Lung adenocarcinoma, n=38 | 0.026315789473684216 | 0.6842105263157896 | 0.2894736842105264 |
| Lung squamous cell carcinoma, n=16 | 0.0 | 0.5625 | 0.43750000000000017 |
| Kidney, n=22 | 0.0 | 0.22727272727272727 | 0.772727272727273 |
| Pancreas, n=22 | 0.0 | 0.09090909090909104 | 0.9090909090909091 |
| Liver, n=31 | 0.0 | 0.4516129032258068 | 0.5483870967741936 |
| Total bladder, n=49 | 0.16 | 0.52 | 0.3200000000000002 |
| ◦ Superficial urothelial carcinoma, n=25 | 0.2800000000000001 | 0.5599999999999999 | 0.16 |
| ◦ Invasive urothelial carcinoma, n=24 | 0.041666666666666664 | 0.45833333333333326 | 0.5 |
| Stomach, n=29 | 0.0 | 0.6896551724137939 | 0.3103448275862071 |
| Total glioma, n=50 | 0.12000000000000002 | 0.54 | 0.34 |
| ◦ Low grade glioma (grade II), n=25 | 0.04000000000000002 | 0.48000000000000015 | 0.48000000000000015 |
| ◦ Glioblastoma (grade IV), n=25 | 0.2 | 0.6000000000000003 | 0.2 |
| Total breast, n=96 | 0.0520833333333334 | 0.8020833333333336 | 0.14583333333333345 |
| ◦ Triple negative breast carcinoma, n=24 | 0.125 | 0.5833333333333333 | 0.2916666666666669 |
| ◦ HR-ERBB2+ breast carcinoma, n=24 | 0.0 | 0.8333333333333337 | 0.16666666666666666 |
| ◦ HR+ ERBB2- breast carcinoma, n=24 | 0.041666666666666664 | 0.9583333333333337 | 0.0 |
| ◦ HR+ ERBB2+ breast carcinoma, n=24 | 0.041666666666666664 | 0.8333333333333337 | 0.125 |Underexpression
Normal expression
Overexpression
