# Supplementary material for: High Positive Correlations between ANRIL and p16-CDKN2A/p15-CDKN2B/p14-ARF Gene Cluster Overexpression in Multi-Tumor Types Suggest Deregulated Activation of an ANRIL–ARF Bidirectional Promoter
Source: Noncoding RNA. 2019 Aug 21;5(3):44. doi: 10.3390/ncrna5030044 (PMC6789474; doi:10.3390/ncrna5030044)
Supplement: Supplementary file 1 [file ncrna-05-00044-s001.zip › Supplemental Figure 2E ANRIL Drak Alsibai et al.pptx]

## Slide 1
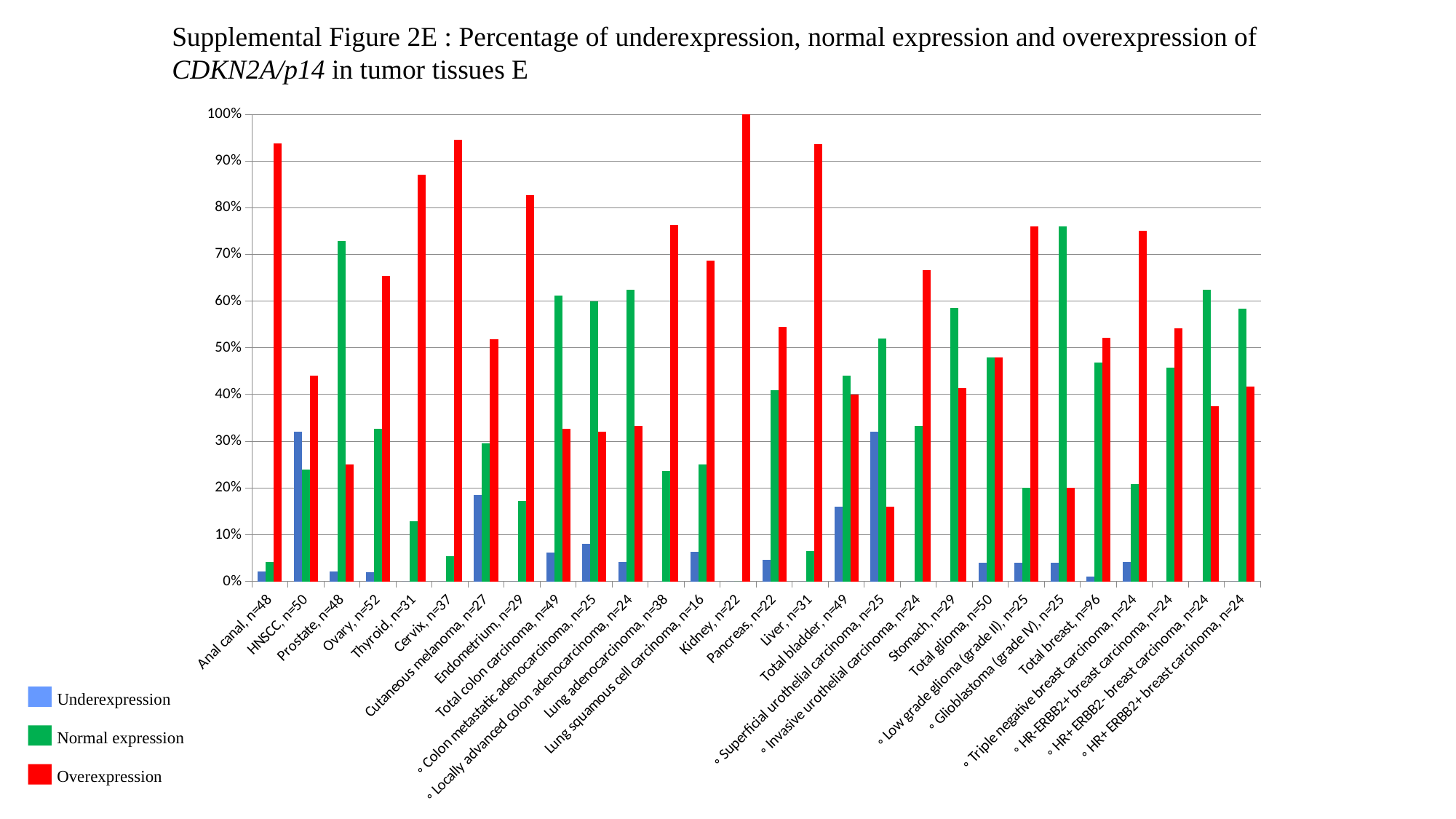

Supplemental Figure 2E : Percentage of underexpression, normal expression and overexpression of CDKN2A/p14 in tumor tissues E
### Chart
| Category | Underexpression | % expr normale | Overexpression |
|---|---|---|---|
| Anal canal, n=48 | 0.02083333333333335 | 0.04166666666666663 | 0.9375 |
| HNSCC, n=50 | 0.3200000000000002 | 0.24000000000000007 | 0.44 |
| Prostate, n=48 | 0.02083333333333335 | 0.7291666666666665 | 0.25 |
| Ovary, n=52 | 0.019230769230769253 | 0.32692307692307715 | 0.6538461538461539 |
| Thyroid, n=31 | 0.0 | 0.12903225806451613 | 0.8709677419354839 |
| Cervix, n=37 | 0.0 | 0.054054054054054085 | 0.9459459459459456 |
| Cutaneous melanoma, n=27 | 0.18518518518518529 | 0.2962962962962963 | 0.5185185185185186 |
| Endometrium, n=29 | 0.0 | 0.17241379310344843 | 0.8275862068965515 |
| Total colon carcinoma, n=49 | 0.06122448979591837 | 0.6122448979591844 | 0.32653061224489827 |
| ◦ Colon metastatic adenocarcinoma, n=25 | 0.08000000000000004 | 0.6000000000000003 | 0.3200000000000002 |
| ◦ Locally advanced colon adenocarcinoma, n=24 | 0.041666666666666664 | 0.6250000000000003 | 0.3333333333333333 |
| Lung adenocarcinoma, n=38 | 0.0 | 0.23684210526315785 | 0.7631578947368421 |
| Lung squamous cell carcinoma, n=16 | 0.0625 | 0.25 | 0.6875 |
| Kidney, n=22 | 0.0 | 0.0 | 1.0 |
| Pancreas, n=22 | 0.04545454545454546 | 0.4090909090909094 | 0.5454545454545454 |
| Liver, n=31 | 0.0 | 0.06451612903225813 | 0.9354838709677415 |
| Total bladder, n=49 | 0.16 | 0.44000000000000006 | 0.4 |
| ◦ Superficial urothelial carcinoma, n=25 | 0.3200000000000002 | 0.52 | 0.16 |
| ◦ Invasive urothelial carcinoma, n=24 | 0.0 | 0.33333333333333337 | 0.6666666666666666 |
| Stomach, n=29 | 0.0 | 0.5862068965517242 | 0.41379310344827575 |
| Total glioma, n=50 | 0.04000000000000002 | 0.48000000000000015 | 0.48000000000000015 |
| ◦ Low grade glioma (grade II), n=25 | 0.04000000000000002 | 0.2 | 0.7600000000000003 |
| ◦ Glioblastoma (grade IV), n=25 | 0.04000000000000002 | 0.7600000000000003 | 0.2 |
| Total breast, n=96 | 0.010416666666666666 | 0.46875 | 0.5208333333333337 |
| ◦ Triple negative breast carcinoma, n=24 | 0.041666666666666664 | 0.20833333333333348 | 0.7500000000000003 |
| ◦ HR-ERBB2+ breast carcinoma, n=24 | 0.0 | 0.45833333333333326 | 0.5416666666666665 |
| ◦ HR+ ERBB2- breast carcinoma, n=24 | 0.0 | 0.6250000000000003 | 0.37500000000000017 |
| ◦ HR+ ERBB2+ breast carcinoma, n=24 | 0.0 | 0.5833333333333333 | 0.4166666666666669 |Underexpression
Normal expression
Overexpression
