# Supplementary material for: High Positive Correlations between ANRIL and p16-CDKN2A/p15-CDKN2B/p14-ARF Gene Cluster Overexpression in Multi-Tumor Types Suggest Deregulated Activation of an ANRIL–ARF Bidirectional Promoter
Source: Noncoding RNA. 2019 Aug 21;5(3):44. doi: 10.3390/ncrna5030044 (PMC6789474; doi:10.3390/ncrna5030044)
Supplement: Supplementary file 1 [file ncrna-05-00044-s001.zip › Supplemental Figure 2A ANRIL Drak Alsibai et al.pptx]

## Slide 1
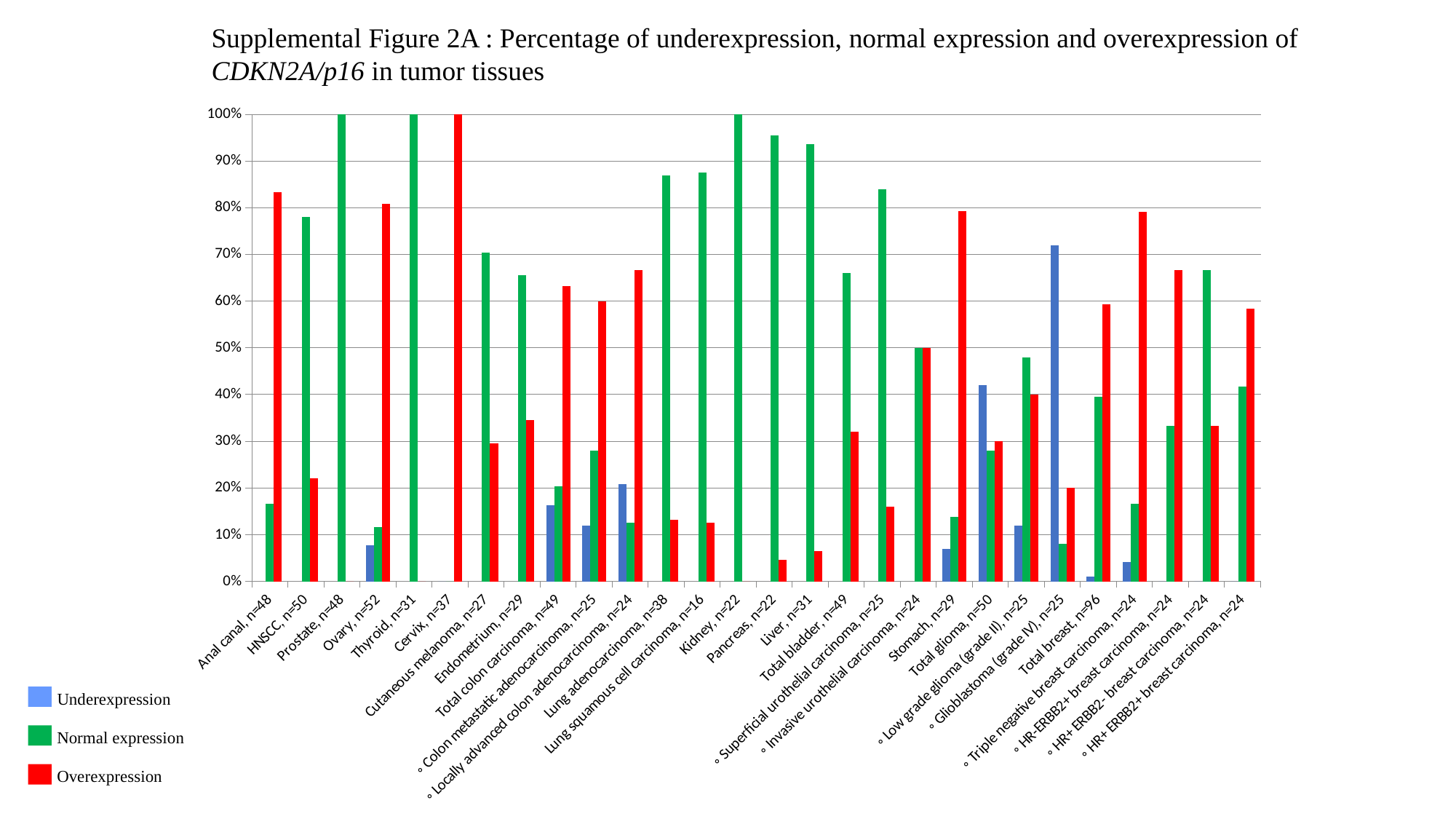

Supplemental Figure 2A : Percentage of underexpression, normal expression and overexpression of CDKN2A/p16 in tumor tissues
### Chart
| Category | Underexpression | % expr normale | Overexpression |
|---|---|---|---|
| Anal canal, n=48 | None | 0.1666666666666669 | 0.8333333333333337 |
| HNSCC, n=50 | None | 0.78 | 0.22 |
| Prostate, n=48 | None | 1.0 | 0.0 |
| Ovary, n=52 | 0.07692307692307693 | 0.11538461538461542 | 0.8076923076923077 |
| Thyroid, n=31 | None | 1.0 | 0.0 |
| Cervix, n=37 | 0.0 | 0.0 | 1.0 |
| Cutaneous melanoma, n=27 | None | 0.7037037037037037 | 0.2962962962962963 |
| Endometrium, n=29 | None | 0.655172413793104 | 0.34482758620689685 |
| Total colon carcinoma, n=49 | 0.16326530612244913 | 0.20408163265306123 | 0.6326530612244902 |
| ◦ Colon metastatic adenocarcinoma, n=25 | 0.12000000000000002 | 0.2800000000000001 | 0.6000000000000003 |
| ◦ Locally advanced colon adenocarcinoma, n=24 | 0.20833333333333345 | 0.125 | 0.6666666666666666 |
| Lung adenocarcinoma, n=38 | None | 0.8684210526315799 | 0.13157894736842113 |
| Lung squamous cell carcinoma, n=16 | None | 0.8750000000000003 | 0.125 |
| Kidney, n=22 | None | 1.0 | 0.0 |
| Pancreas, n=22 | None | 0.954545454545455 | 0.04545454545454546 |
| Liver, n=31 | None | 0.9354838709677415 | 0.0645161290322581 |
| Total bladder, n=49 | None | 0.6600000000000004 | 0.3200000000000002 |
| ◦ Superficial urothelial carcinoma, n=25 | None | 0.8400000000000003 | 0.16 |
| ◦ Invasive urothelial carcinoma, n=24 | None | 0.5 | 0.5 |
| Stomach, n=29 | 0.06896551724137931 | 0.13793103448275867 | 0.7931034482758621 |
| Total glioma, n=50 | 0.42000000000000015 | 0.2800000000000001 | 0.30000000000000016 |
| ◦ Low grade glioma (grade II), n=25 | 0.12000000000000002 | 0.48000000000000015 | 0.4 |
| ◦ Glioblastoma (grade IV), n=25 | 0.7200000000000003 | 0.08000000000000011 | 0.2 |
| Total breast, n=96 | 0.010416666666666666 | 0.39583333333333337 | 0.59375 |
| ◦ Triple negative breast carcinoma, n=24 | 0.041666666666666664 | 0.16666666666666669 | 0.7916666666666665 |
| ◦ HR-ERBB2+ breast carcinoma, n=24 | 0.0 | 0.33333333333333337 | 0.6666666666666666 |
| ◦ HR+ ERBB2- breast carcinoma, n=24 | 0.0 | 0.6666666666666667 | 0.3333333333333333 |
| ◦ HR+ ERBB2+ breast carcinoma, n=24 | 0.0 | 0.4166666666666669 | 0.5833333333333334 |Underexpression
Normal expression
Overexpression
