# Supplementary material for: High Positive Correlations between ANRIL and p16-CDKN2A/p15-CDKN2B/p14-ARF Gene Cluster Overexpression in Multi-Tumor Types Suggest Deregulated Activation of an ANRIL–ARF Bidirectional Promoter
Source: Noncoding RNA. 2019 Aug 21;5(3):44. doi: 10.3390/ncrna5030044 (PMC6789474; doi:10.3390/ncrna5030044)
Supplement: Supplementary file 1 [file ncrna-05-00044-s001.zip › Informed Consent Paper of Curie Institute.pdf]

Exemplaire à compléter et à remettre à l'hôpital.

Consentements

pour **la collecte, le stockage et l'utilisation de mes données** et de **mes échantillons biologiques** à des fins de **recherche en cancérologie**

institutCurie

Je, soussigné(e) :

Nom de naissance ....

Nom marital..

Prénom ....

Date de naissance ..../........../.........

N° de dossier (N° à 7 chiffres) ☐☐☐☐☐☐☐

I. INFORMATION POUR LA NON OPPOSITION

Je reconnais avoir lu et compris ce qui m'a été préalablement exposé sur la collecte, le stockage et l'utilisation de mes données personnelles pour des recherches scientifiques en cancérologie, et que je dispose d'un droit d'opposition au traitement de mes données personnelles dans les conditions définies par l'Article 21 6 du Règlement Général sur la Protection des Données.

II. CONSENTEMENTS

Je coche les cases correspondant à mes choix :

Données personnelles

Consentement n°1 : pour l'utilisation de mes données personnelles utiles à l'interprétation des résultats des examens génétiques réalisés à partir de mes prélèvements sanguins, salivaires ou sur la lésion tumorale pour laquelle je suis ou j'ai été traité(é)

J'accepte que l'Institut Curie utilise mes données personnelles pour l'examen de mes caractéristiques génétiques à des fins de recherches en cancérologie.

☐ oui

☐ non

(En cas d'accord, je comprends que les données enregistrées à l'occasion des recherches comportant des données génétiques font l'objet d'un traitement informatisé.)

Echantillons biologiques

Consentement n°2 : pour le stockage et l'utilisation éventuelle de mes échantillons biologiques

J'accepte que mes échantillons, recueillis lors des soins, soient stockés et utilisés dans le cadre de programmes de recherche en cancérologie :

☐ oui

☐ non

Consentement n°3 : pour l'examen éventuel de mes caractéristiques génétiques

J'accepte que les caractéristiques génétiques issues de mes prélèvements biologiques puissent être analysées dans le cadre de recherches dans le domaine de la cancérologie :

☐ oui

☐ non

Consentement n°3 bis : pour le retour d'informations issues de ces éventuelles études génétiques ***(à compléter en cas d'accord du consentement n°3)***

Suite à l'examen possible de mes caractéristiques génétiques pour la recherche, des informations importantes concernant ma santé, validées sur le plan médical et pouvant conduire à des mesures de prévention ou de soins, que ce soit dans le domaine de la cancérologie ou dans un autre domaine, pourraient être identifiées.

Dans cette éventualité, **j'accepte que** ces informations me soient communiquées,
(par l'intermédiaire de mon médecin référent à l'Institut Curie) :

☐ oui

☐ non

Consentement n°4 : pour un prélèvement unique et l'utilisation de deux tubes de mon sang ou d'un prélèvement salivaire ***(à compléter si acceptation du consentement n°3)***

Dans le cadre de mon parcours de soin, **j'accepte qu'un** prélèvement de deux tubes de sang ou, sous certaines conditions, d'un prélèvement salivaire puisse être réalisé pour une finalité de recherche :

☐ oui

☐ non

J'ai bien conscience que je peux retirer mes consentements, ou une partie d'entre eux, à tout moment, sans aucune conséquence sur la qualité et la continuité de mes soins.

J'ai bien noté que c'est également à tout moment que je peux exercer mes droits d'accès, de rectification et d'opposition, d'effacement, de portabilité à mes données personnelles et d'introduire une réclamation auprès d'une autorité de contrôle. De même, je peux vérifier l'identité du responsable du traitement, la base juridique, la finalité du traitement et les destinataires des données ainsi que la durée de conservation auprès du DPO à l'adresse suivante : DPO@curie.fr

J'ai vérifié que les cases cochées correspondent bien à mes choix.

Fait à ...., le…………………………………..

Signature :

Si vous souhaitez plus d'information sur la conduite des recherches ou si vous changez d'avis sur vos choix, vous pouvez adresser un courrier ou un e-mail en indiquant vos nouvelles décisions à l'une des adresses suivantes :

Gestionnaire du Centre de Ressources Biologiques
Institut Curie - 26, rue d'Ulm
75248 PARIS CEDEX 05

consentement@curie.fr
