# Supplementary figures and images for: High Positive Correlations between ANRIL and p16-CDKN2A/p15-CDKN2B/p14-ARF Gene Cluster Overexpression in Multi-Tumor Types Suggest Deregulated Activation of an ANRIL–ARF Bidirectional Promoter
Source: Noncoding RNA. 2019 Aug 21;5(3):44. doi: 10.3390/ncrna5030044 (PMC6789474; doi:10.3390/ncrna5030044)

## Slide 1
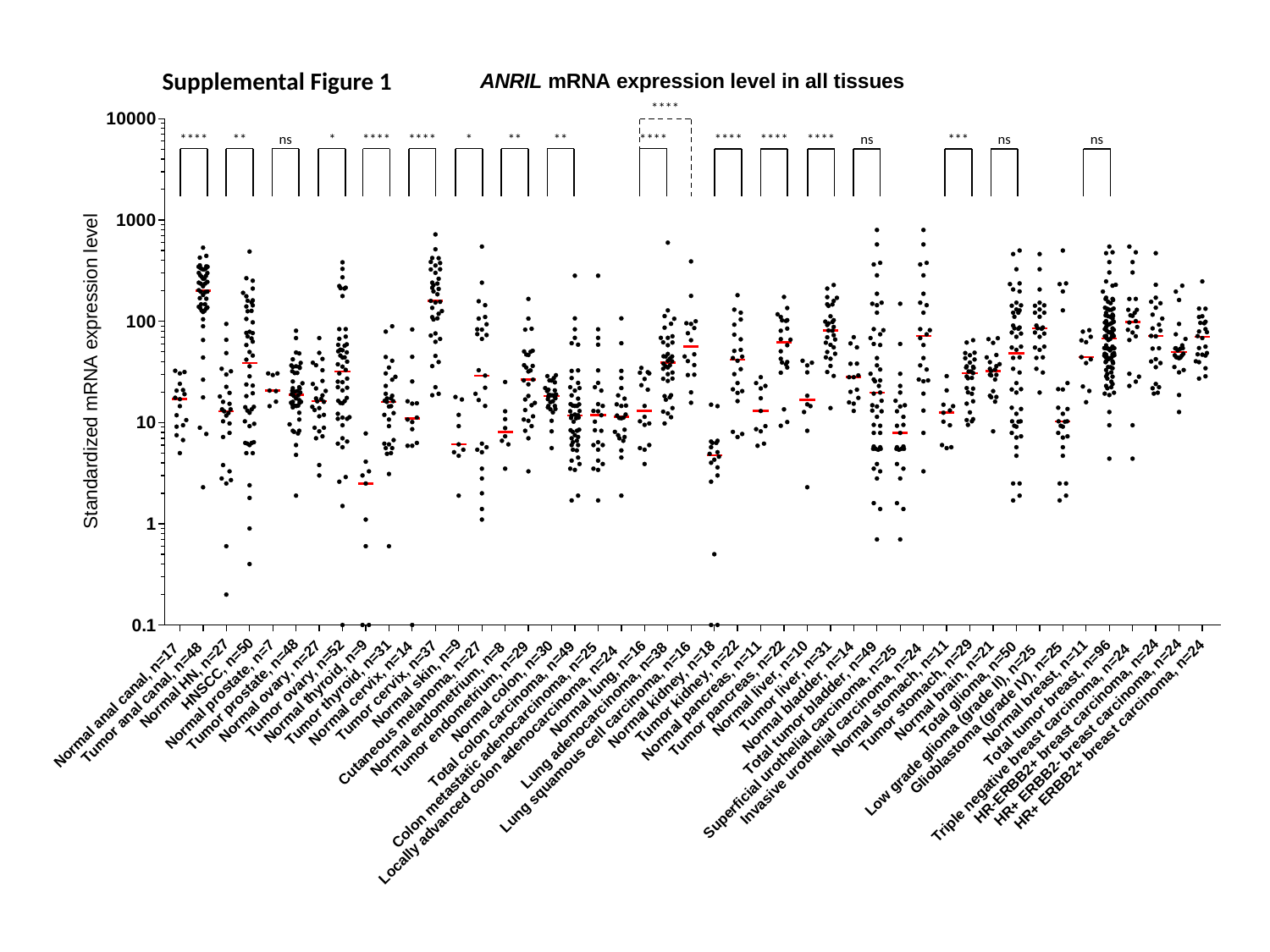

Supplemental Figure 1
****
****
**
ns
*
****
****
*
**
**
****
****
****
****
ns
***
ns
ns

Supplement: Supplementary file 1 [file ncrna-05-00044-s001.zip › Supplemental Figure 1 ANRIL Drak Alsibai et al.pptx]

## Slide 1
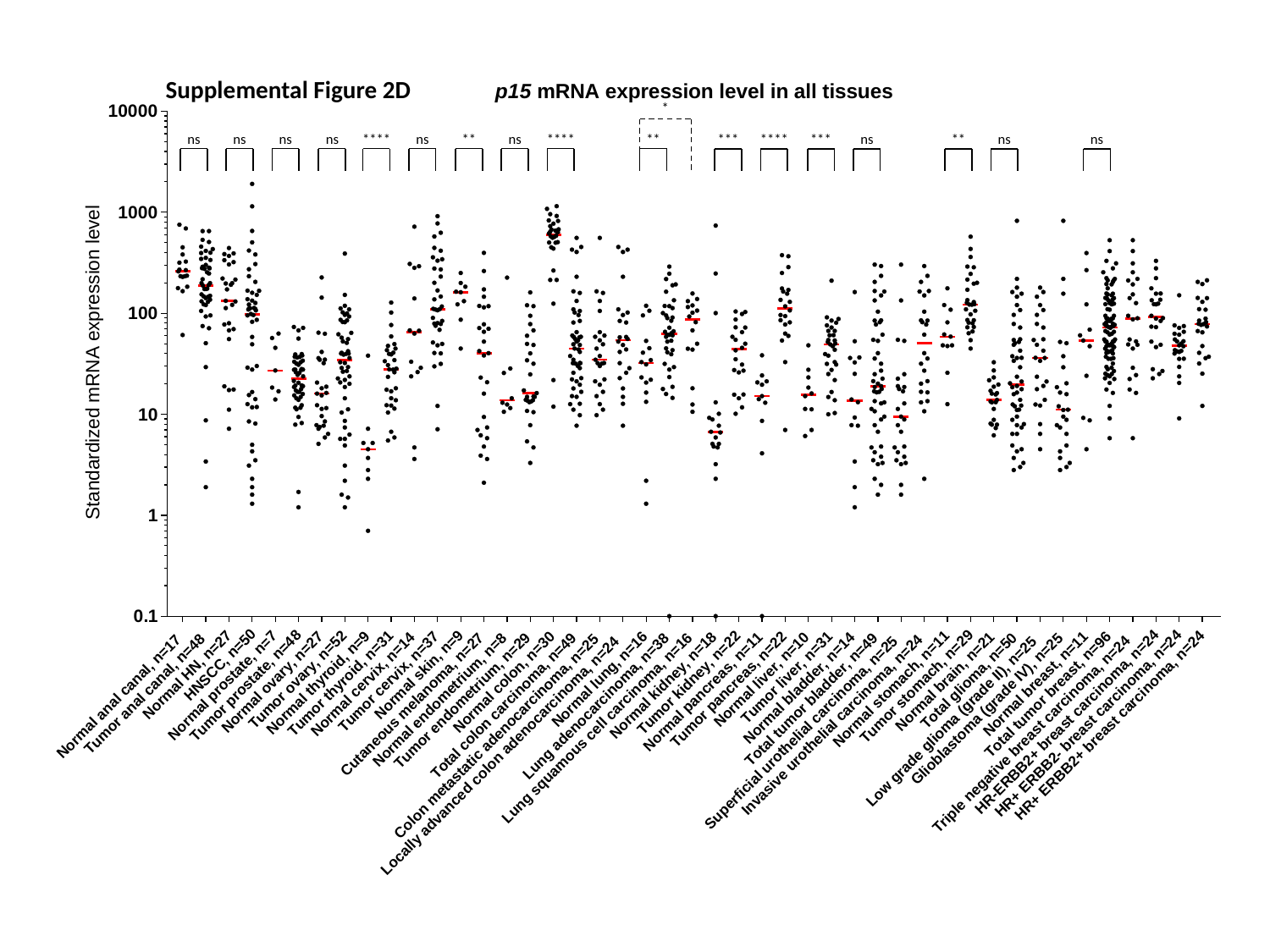

Supplemental Figure 2D
*
ns
ns
ns
ns
****
ns
**
ns
****
**
***
****
***
ns
**
ns
ns

Supplement: Supplementary file 1 [file ncrna-05-00044-s001.zip › Supplemental Figure 2D ANRIL Drak Alsibai et al.pptx]

## Slide 1
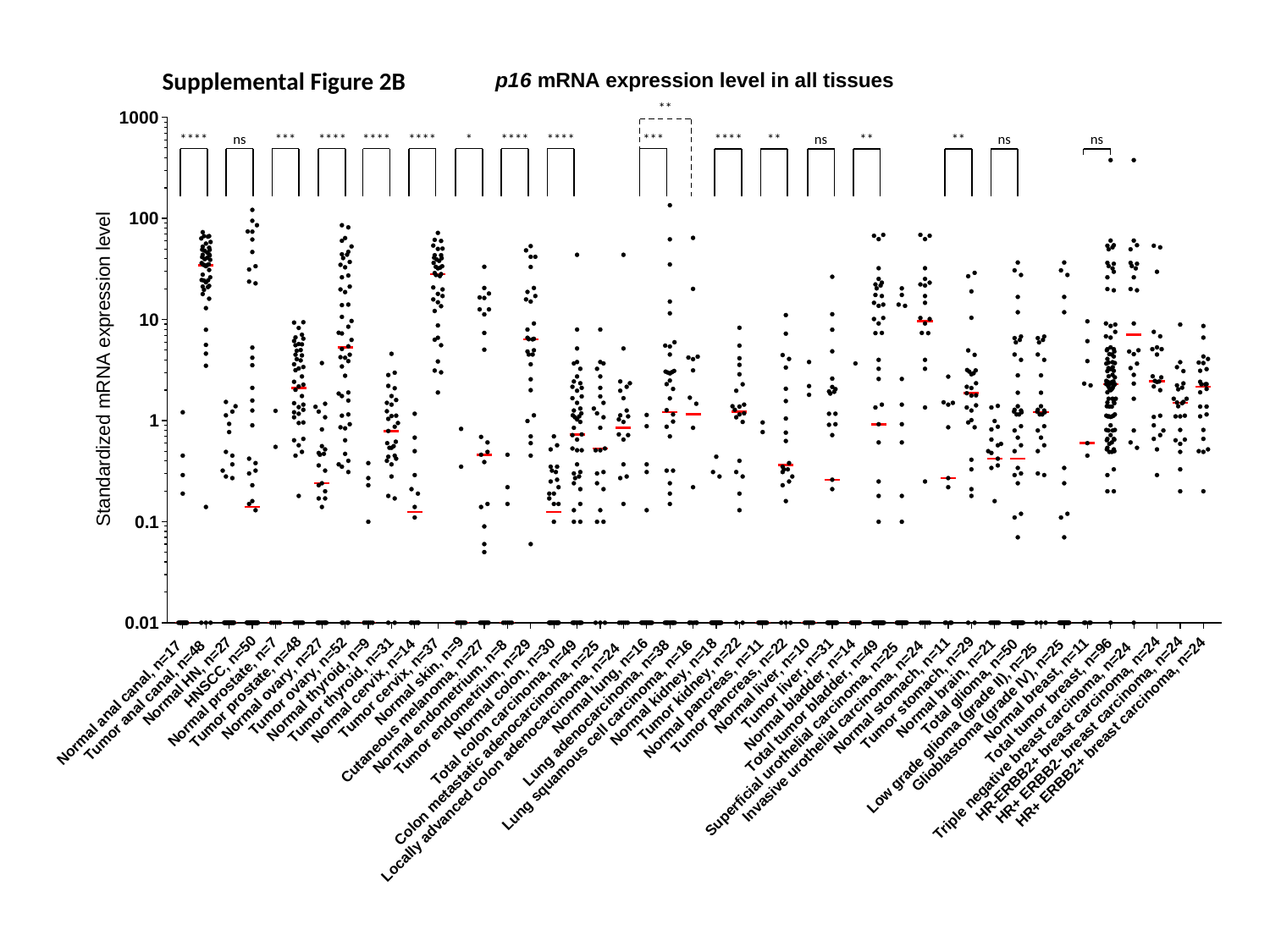

Supplemental Figure 2B
**
****
ns
***
****
****
****
*
****
****
***
****
**
ns
**
**
ns
ns

Supplement: Supplementary file 1 [file ncrna-05-00044-s001.zip › Supplemental Figure 2B ANRIL Drak Alsibai et al.pptx]

## Slide 1
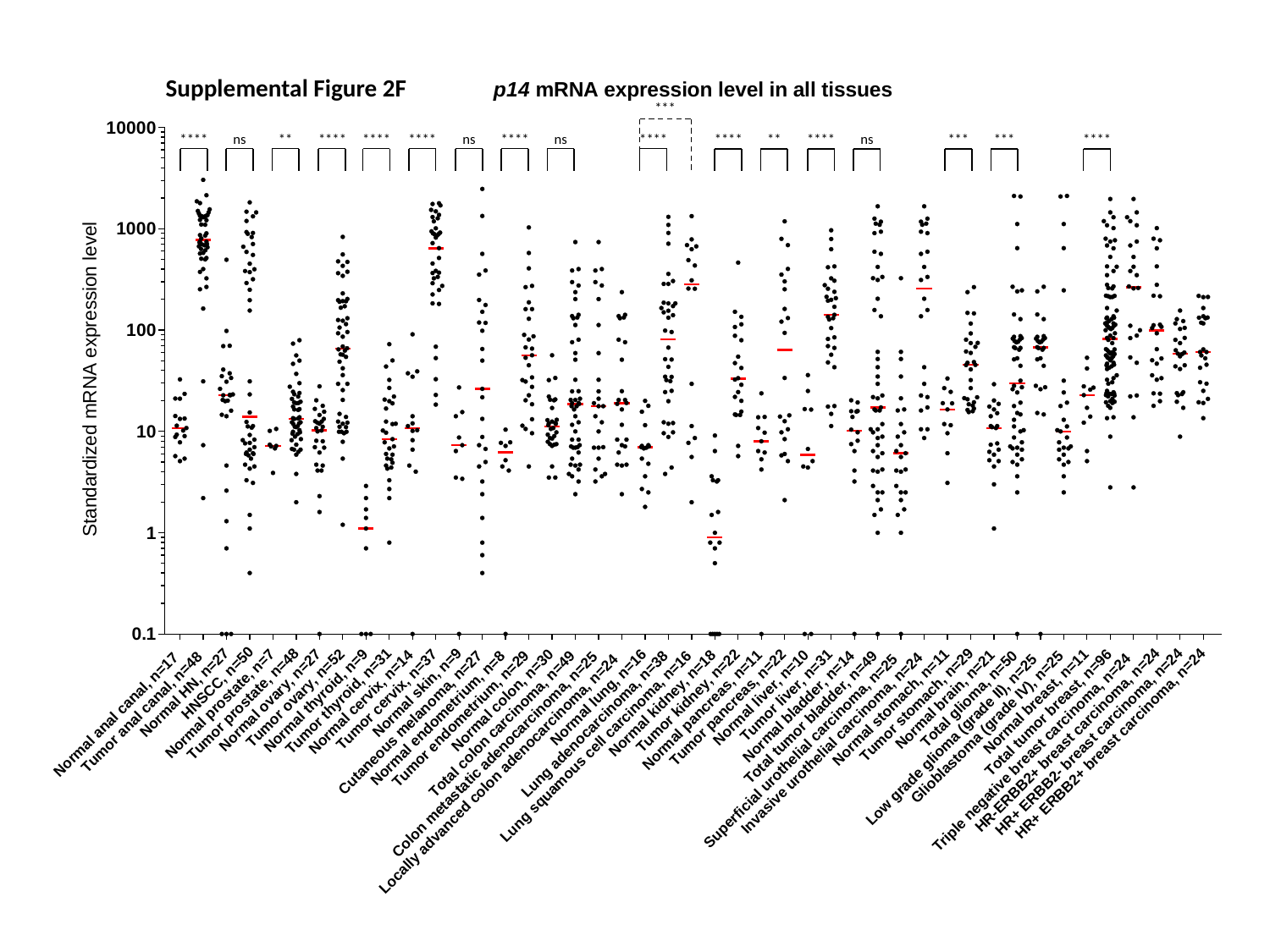

Supplemental Figure 2F
***
****
ns
**
****
****
****
ns
****
ns
****
****
**
****
ns
***
***
****

Supplement: Supplementary file 1 [file ncrna-05-00044-s001.zip › Supplemental Figure 2F ANRIL Drak Alsibai et al.pptx]
